# Quadrilateral Pinwheel Flap Reconstruction for a Complex Colocutaneous Fistula-Associated Flank Wound in a Paraplegic Patient: A Case Report

**DOI:** 10.3390/jcm15062394

**Published:** 2026-03-20

**Authors:** Joon Hyuk Lee, Tae Gon Kim

**Affiliations:** Department of Plastic and Reconstructive Surgery, College of Medicine, Yeungnam University, 170 Hyeonchung-ro, Nam-gu, Daegu 42415, Republic of Korea; mericle@naver.com

**Keywords:** paraplegia, colonic fistula, surgical flaps, wound healing, negative-pressure wound therapy

## Abstract

**Background/Objectives:** Chronic wounds are a major source of morbidity in patients with paraplegia, often resulting in repeated treatment, prolonged hospitalization, and reduced quality of life. Reconstruction becomes particularly challenging when a wound arises in a scarred trunk region and is further complicated by deep infection, osteomyelitis, or enteric fistula. We describe the staged management of a complex left flank wound in a paraplegic patient, initially reconstructed with a quadrilateral pinwheel flap and later requiring multidisciplinary salvage for recurrence associated with rib osteomyelitis and a colocutaneous fistula. **Methods:** A paraplegic man in his 50s presented with a chronic left flank wound after repeated full-thickness skin graft failure and persistent Pseudomonas aeruginosa infection. After wide debridement, the approximately 7 × 7 cm defect was reconstructed with a quadrilateral pinwheel flap composed of four Limberg-style rhomboid fasciocutaneous flaps positioned at the 12, 3, 6, and 9 o’clock orientations, elevated at the level of the deep fascia, and transposed into the central defect, with adjunctive negative-pressure wound therapy (NPWT). Approximately 1 year later, recurrence with rib osteomyelitis required rib resection. During NPWT, feculent drainage led to the diagnosis of a colocutaneous fistula. Subsequent multidisciplinary treatment included fistula tract resection, colonic repair with omental patching, transposition of vascularized omentum into the chest wall cavity to obliterate dead space, continued NPWT, and delayed primary closure. **Results:** Initial local flap reconstruction achieved wound coverage, and immediate postoperative clinical assessment, including pinprick and refill testing, confirmed satisfactory flap perfusion; however, delayed recurrence developed in association with rib osteomyelitis. After definitive fistula surgery, dead-space management with vascularized omentum, wound conditioning with staged NPWT, and delayed primary closure, the wound healed completely. At 6 months after delayed closure, no recurrence of fistula, osteomyelitis, wound dehiscence, or soft-tissue breakdown was observed, and the patient’s daily comfort and functional independence were improved compared with the preoperative condition. **Conclusions**: A quadrilateral pinwheel flap may provide an effective tension-dispersing local fasciocutaneous option for selected scarred trunk defects in high-risk patients. However, when chronic wounds are compounded by deep infection and enteric fistula, durable healing depends not on flap design alone but on staged multidisciplinary management incorporating definitive source control, vascularized tissue transfer for dead-space elimination, NPWT, and appropriately timed closure.

## 1. Introduction

Chronic wounds are a major source of morbidity in patients with spinal cord injury and paraplegia. In this population, recurrent soft-tissue breakdown often leads to repeated hospitalization, prolonged treatment, and substantial loss of quality of life and functional independence [1]. Sensory loss, immobility, altered pressure distribution, repeated contamination, and compromised local tissue quality make these patients particularly susceptible to chronic and recurrent wounds [1]. Once tissue breakdown occurs, healing is often delayed by bacterial colonization, persistent inflammation, malnutrition, repeated operative intervention, and fibrosis of the surrounding soft tissues [1].

Trunk wounds are especially difficult to manage when they arise in scarred or previously operated fields. The flank region is exposed to pressure, shear stress during transfers and repositioning, and contamination from adjacent structures. In addition, chronic inflammation, prior grafting, and repeated wound breakdown can render the surrounding tissue stiff and inelastic. Under these conditions, skin grafting alone is often insufficient because it does not address persistent infection, poor wound-bed vascularity, or a residual cavity (dead space). Chronic wounds are increasingly understood as biologically dysregulated microenvironments characterized by prolonged inflammation, oxidative stress, hypoxia, impaired angiogenesis, and defective transition from inflammation to proliferation. Durable repair therefore depends not only on surface closure but also on restoration of a biologically favorable local environment [2,3].

Enterocutaneous and colocutaneous fistulas further increase the complexity of management. These fistulas can cause ongoing wound contamination, sepsis, electrolyte imbalance, malnutrition, and recurrent soft-tissue breakdown, thereby making definitive closure difficult [4,5]. Their successful treatment requires staged management focused on infection control, wound bed optimization, correction of systemic derangements, and appropriately timed definitive surgery [4,5]. In wounds complicated by osteomyelitis and dead space, reconstructive planning must go beyond simple coverage and prioritize durable source control and biologically favorable tissue support.

For selected trunk defects, local fasciocutaneous flaps remain attractive because they avoid the additional donor-site morbidity and operative burden associated with muscle flaps or free tissue transfer. The rhomboid (Limberg) flap is a versatile transposition flap that recruits adjacent tissue with reliable vascularity and geometric flexibility [6,7]. Modified pinwheel designs extend this principle by distributing closure tension across several vectors rather than concentrating it in a single transposition direction [8,9]. This multi-vector design may be advantageous in scarred wounds where tissue mobility is limited and a single large transposition flap is difficult to design safely. Published experience with pinwheel-type flaps, however, has been largely limited to scalp and temporal defects [8,9]. Evidence supporting their use in contaminated trunk wounds, particularly those associated with fistula or osteomyelitis, remains very limited.

Accordingly, this case report was undertaken to address an evident clinical gap. We present a paraplegic patient with a chronic left flank wound initially reconstructed with a quadrilateral pinwheel flap and later complicated by recurrence, rib osteomyelitis, and a colocutaneous fistula. This case highlights both the practical role of a multi-vector local fasciocutaneous flap in a scarred trunk defect and the importance of staged multidisciplinary management when deeper infection and fistulous contamination are present.

## 2. Case Presentation

### 2.1. Initial Presentation and Primary Reconstruction

A paraplegic man in his 50s was referred to our department for management of a chronic wound in the left flank region. The wound had developed after skin necrosis at the site of a previously applied procedural bandage. At another institution, three attempts at full-thickness skin grafting had failed in the setting of persistent Pseudomonas aeruginosa infection, resulting in a chronically draining wound in a scarred field.

**Figure 1:**
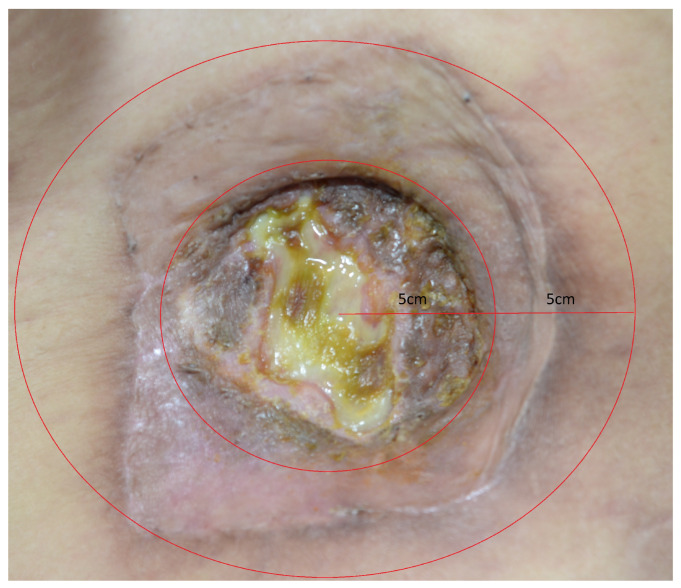
Preoperative wound in the left flank region. A central approximately 5 × 5 cm defect is present within an approximately 10 × 10 cm atrophic graft scar; these measurements were based on clinical examination and operative assessment. Red guideline markings indicate the central wound defect and the surrounding atrophic scar.

At presentation, physical examination revealed an approximately 10 × 10 cm atrophic graft scar in the left flank region with a central 5 × 5 cm defect (Figure 1). These dimensions were based on clinical examination and operative assessment rather than scale-calibrated photographic measurement. The wound was characterized by chronic granulation tissue and serous discharge. The surrounding tissues were fibrotic and atrophic, suggesting poor elasticity and limited capacity for tension-free direct closure. Given the chronic draining wound, repeated graft failure, persistent infection, and poor local tissue quality, we judged that definitive reconstruction would require wide debridement followed by vascularized tissue coverage rather than another grafting procedure.

### 2.2. Primary Reconstruction with a Quadrilateral Pinwheel Flap

Under general anesthesia, wide excision of the unstable scar, chronic granulation tissue, and nonviable soft tissue was performed. Following debridement, the resulting defect measured approximately 7 × 7 cm and involved full-thickness soft tissue.

Because the defect lay in a fibrotic, previously grafted field with limited tissue laxity, we selected a quadrilateral pinwheel flap to recruit adjacent tissue from multiple directions and distribute closure tension across several shorter vectors. A more extensive reconstructive option, such as a muscle flap or free tissue transfer, was not selected at this stage because the defect appeared reconstructable with local tissue recruitment after adequate debridement, and avoiding additional donor-site morbidity and operative burden was preferable in this high-risk patient.

**Figure 2:**
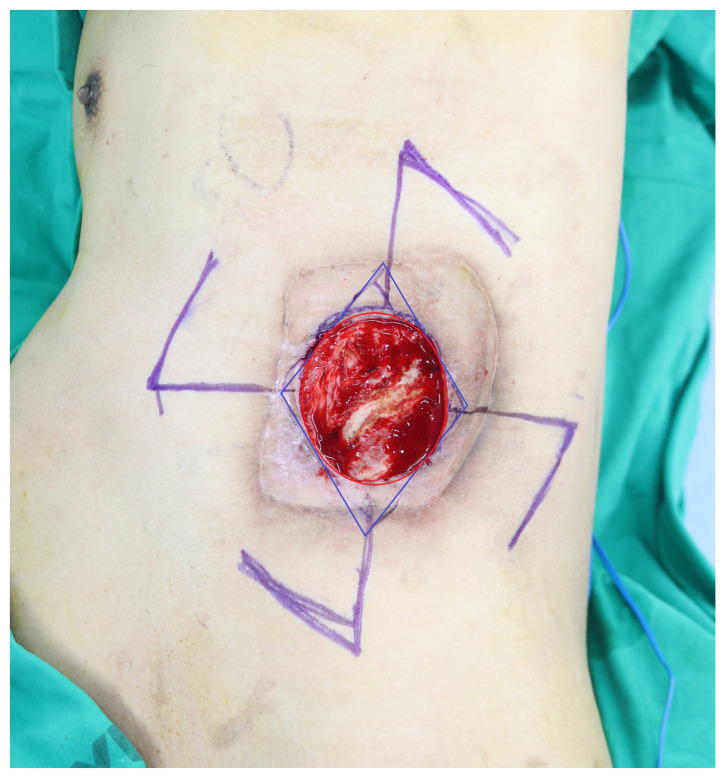
Quadrilateral pinwheel flap design. Four Limberg-style rhomboid fasciocutaneous flaps are outlined at the 12, 3, 6, and 9 o’clock positions around the central defect. Blue lines indicate the boundaries of the rhomboid flaps, and red lines indicate the defect margin.

The quadrilateral pinwheel flap was designed using four Limberg-style rhomboid fasciocutaneous flaps positioned at the 12, 3, 6, and 9 o’clock orientations (Figure 2). This local fasciocutaneous design relied on adjacent tissue perfusion through the subfascial/subdermal plexus rather than on individually dissected named perforators.

**Figure 3:**
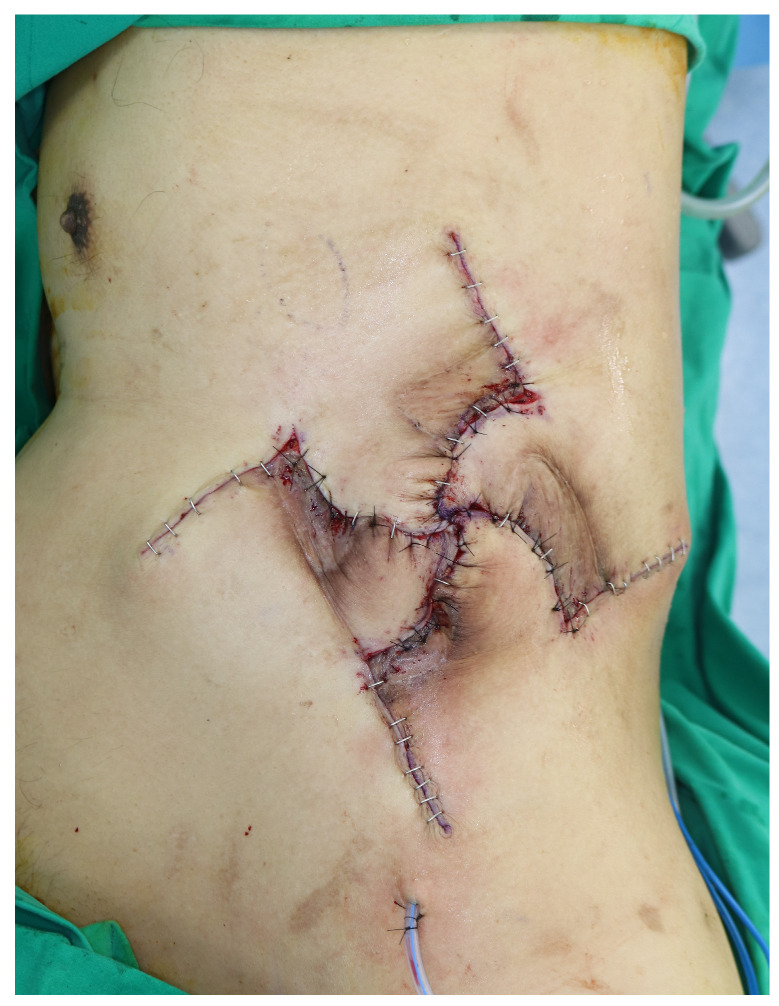
Immediate postoperative flap inset. The four transposed flaps provide centralized coverage with balanced tension distribution across multiple wound vectors and aligned wound margins. Postoperative clinical assessment, including pinprick and refill testing, confirmed satisfactory flap perfusion.

Each rhomboid flap was elevated in the fasciocutaneous plane over the underlying muscle. The flaps were then transposed into the central defect at approximately 90 degrees to achieve complete coverage with balanced tension distribution across multiple wound vectors rather than concentration of tension along any single wound edge (Figure 3). The deep layer was sutured with 3-0 Vicryl, and the skin was closed using staples and 4-0 nylon sutures. A closed-suction drain was placed. NPWT was then applied over the incision at 125 mmHg, with dressing changes at 3-day intervals, to stabilize the wound environment and support early postoperative wound conditioning.

### 2.3. Early Postoperative Course

The immediate postoperative course was largely uneventful. The flap remained viable and achieved initial wound coverage. Postoperative clinical assessment, including pinprick testing and refill testing, indicated satisfactory perfusion of the transposed fasciocutaneous flaps. On postoperative day 16, minor wound separation developed and was managed with local debridement and secondary closure. No major flap necrosis or vascular compromise occurred, and the patient was discharged without further immediate complications.

This early result suggested that the flap design was technically adequate for initial coverage. The episode of minor dehiscence, however, indicated that the wound bed and surrounding tissues remained biologically fragile despite satisfactory flap perfusion. Because the patient had paraplegia, repeated prior treatment failure, and poor local tissue quality, continued surveillance was considered necessary even after apparently successful early healing.

### 2.4. Late Recurrence with Rib Osteomyelitis

Approximately 1 year after the initial reconstruction, the patient presented again with fever, recurrent wound breakdown, and purulent discharge at the same site. Clinical and imaging findings were consistent with rib osteomyelitis. This indicated that the disease process had progressed beyond the superficial soft tissues and now involved deeper structural infection.

This delayed presentation suggested that the initial reconstructive success had not eliminated the underlying disease process. Thoracic surgery performed resection of the involved ribs, leaving an approximately 10 cm defect within a densely scarred and previously operated field. The wound was irrigated and curetted, and an attempt at primary closure was made. However, early partial dehiscence recurred, indicating that local tissue conditions remained unfavorable. Because the wound failed to remain closed after re-approximation in a scarred and previously operated field, NPWT was selected to control exudate, reduce edema, promote granulation tissue formation, and provide temporary wound stabilization while the possibility of ongoing deep contamination was further evaluated.

### 2.5. Diagnosis of the Colocutaneous Fistula

During NPWT, feculent drainage was observed in the canister and at the wound site, raising suspicion of enteric communication. Contrast imaging confirmed a colocutaneous fistula between the colon and the chest wall wound. This finding substantially altered the treatment strategy, as definitive healing could no longer be expected from local wound management alone. Persistent enteric contamination would continue to undermine any attempt at closure unless the fistula was surgically addressed.

At this point, the clinical focus shifted from local wound control to identification and definitive treatment of the source of contamination. The diagnosis of the fistula also emphasized the importance of reassessing recurrent wounds that fail to progress as expected. In this patient, wound breakdown was not caused solely by poor local tissue quality or superficial infection; it was also driven by ongoing internal contamination through the fistulous tract.

### 2.6. Multidisciplinary Salvage Procedure

Once the fistula was identified, a staged multidisciplinary strategy was adopted involving thoracic surgery, general surgery, and plastic surgery. General surgery performed laparotomy with resection of the fistula tract and primary repair of the involved colon. An omental patch was applied to reinforce the colonic repair, and a vascularized tongue of omentum was transferred into the chest wall cavity to obliterate the residual dead space.

The plastic surgery team then performed additional curettage and irrigation of the wound. Because the wound remained in a recently contaminated field, tight definitive closure was avoided at this stage. Instead, the soft tissues were loosely approximated, and NPWT was re-applied to maintain a controlled wound environment while allowing continued drainage management and observation for persistent contamination.

This staged approach was chosen because successful reconstruction in the setting of fistula and osteomyelitis required more than soft-tissue closure. It required definitive source control, elimination of the residual cavity, and sufficient time for the wound to transition from a contaminated state to a biologically favorable condition before final closure.

### 2.7. Delayed Primary Closure and Outcome

NPWT was continued with scheduled dressing changes. Over time, the wound showed progressive improvement, with healthy granulation tissue and no further feculent or purulent drainage. By postoperative day 19 after fistula repair, the wound was considered sufficiently clean and stable for definitive closure. NPWT was discontinued, and delayed primary closure was performed using interrupted sutures.

**Figure 4:**
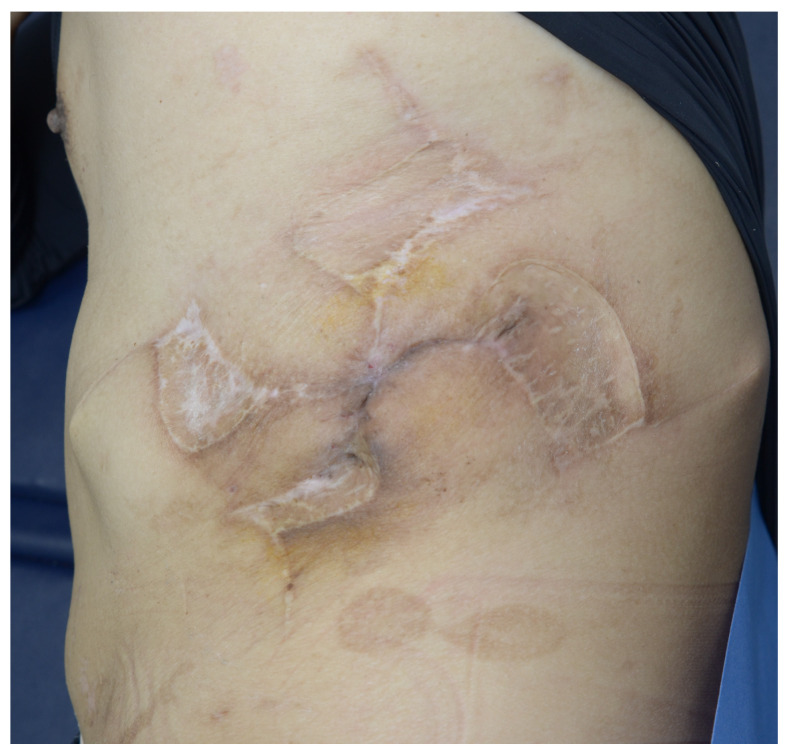
Final postoperative appearance. At 6 months after delayed primary closure, durable healing is observed without recurrent dehiscence, infection, osteomyelitis, or fistula.

At 6 months after delayed primary closure, the wound had healed completely without recurrent infection, osteomyelitis, fistula, or dehiscence (Figure 4). The clinical benefit extended beyond durable wound closure alone: based on follow-up assessment, the patient also showed improvement in daily comfort and functional independence compared with the preoperative condition. The final result suggested that durable healing became possible only after comprehensive management of the underlying pathology, including bowel repair, vascularized dead-space obliteration, staged wound conditioning, and appropriately timed closure.

## 3. Discussion

This case highlights several important principles in the management of chronic wounds in paraplegic patients. First, wound healing failure in this population is usually multifactorial. Mechanical factors such as pressure, shear, and immobility coexist with biological factors such as chronic bacterial burden, repeated contamination, fibrosis, poor tissue perfusion, and diminished wound healing capacity [1]. As a result, even technically adequate reconstruction may fail if the underlying infectious or structural pathology is not recognized and corrected.

Second, the case demonstrates that a quadrilateral pinwheel flap can be a useful local fasciocutaneous option for selected scarred trunk defects. Classical Limberg flaps are well established as versatile transposition flaps for cutaneous defects, and multiple modifications have been described for larger or irregular wounds [6,7,10]. In the present case, the use of four rhomboid-based fasciocutaneous flaps allowed tissue recruitment from multiple directions and dispersed closure forces around a central defect. This configuration was particularly advantageous in a stiff and scarred field where a single larger transposition flap may have concentrated tension and been more vulnerable to compromise.

The pinwheel concept has previously been described primarily for scalp and temporal defects, where tissue mobility is similarly limited [8,9]. The present report suggests that the same biomechanical principle can be adapted to selected trunk wounds. By using shorter transposition components and multiple tension vectors, the design may help achieve a more balanced closure pattern in compromised local tissues.

However, the later recurrence in our patient also demonstrates the limitations of flap design alone. The initial reconstruction achieved coverage, but long-term healing was undermined by deeper pathology, specifically rib osteomyelitis and a colocutaneous fistula. This distinction is clinically important. Recurrent breakdown after apparently successful wound coverage should prompt evaluation for underlying osteomyelitis, dead space, or fistulous communication rather than being attributed solely to superficial wound tension or poor flap choice. The later recurrence in our patient was unlikely to be explained solely by primary flap ischemia, because the early postoperative course demonstrated flap viability, preserved perfusion, and initial wound coverage without major necrosis or vascular compromise. Rather, the delayed failure was more plausibly related to persistent or progressive deep infectious burden and occult internal contamination that were not yet fully anatomically defined at the time of the first reconstruction. Nevertheless, because this was a retrospective single-case report, incomplete eradication of deep bacterial burden during the initial debridement cannot be entirely excluded.

Management of enterocutaneous and colocutaneous fistula requires staged and multidisciplinary care. Established principles include control of sepsis, optimization of nutrition and fluid balance, wound management, anatomical definition of the fistula, and definitive surgical treatment at an appropriate time [4,5]. In our patient, definitive healing was achieved only after fistula tract resection, colonic repair, elimination of contamination, and delayed closure of the wound once the local environment became favorable.

NPWT served as an important adjunct throughout this process. It helped control exudate, reduce edema, promote granulation tissue formation, and maintain a controlled wound environment during both the post-rib resection phase and the post-fistula repair phase. In this case, NPWT did not replace definitive surgery but rather functioned as a bridge strategy supporting staged wound conditioning until definitive closure became feasible.

The use of vascularized omentum also played a central role in achieving durable healing. The omentum is well recognized for its pliability, vascularity, immunologic activity, and ability to conform to irregular cavities. These features make it particularly valuable in contaminated fields and in defects associated with dead space after debridement or rib resection [11]. In the present case, the omentum reinforced the colonic repair and simultaneously obliterated the chest wall cavity, thereby addressing one of the key structural causes of persistent wound failure.

From a clinical perspective, the present case is instructive not only because it demonstrates adaptation of a quadrilateral pinwheel flap to a scarred flank defect, but also because it shows that flap design alone was insufficient once deeper pathology became clinically relevant. The reconstructive lesson, therefore, is twofold: a local multi-vector fasciocutaneous flap can provide effective initial coverage in a scarred field, but durable healing in contaminated and recurrent wounds depends on timely reassessment, recognition of occult deeper causes, and staged multidisciplinary source control.

This report has limitations. It describes a single case and cannot establish general indications or comparative superiority over other reconstructive options such as muscle flaps, perforator flaps, or free tissue transfer. In addition, the final outcome reflected the cumulative effect of multiple interventions rather than the quadrilateral pinwheel flap alone. Nevertheless, this case remains instructive because it demonstrates a practical local flap design for a difficult scarred trunk defect and emphasizes the importance of investigating deeper causes when recurrence occurs after apparently adequate surface reconstruction. In addition, formal patient-reported outcome measures and validated functional assessments were not prospectively collected, and therefore the present report cannot comprehensively quantify changes in quality of life, sitting tolerance, transfer-related function, or wound care burden, even though the patient showed clinical improvement in daily comfort and functional independence after definitive healing. Similarly, highly specific intraoperative geometric details, such as the exact dimensions of each rhomboid flap, were not prospectively documented in the operative record.

Future studies involving a larger case series would be useful to clarify the indications for multi-vector local flap designs in trunk reconstruction, particularly in patients with scarred tissue or prior graft failure. It would also be valuable to compare outcomes among local fasciocutaneous flaps, muscle flaps, and more complex reconstructive strategies in patients with chronic contaminated wounds and major comorbidities.

## 4. Conclusions

The central lesson of this case is that successful treatment of a chronic paraplegia-associated flank wound required both an appropriate local flap strategy and definitive management of the underlying source of failure. A quadrilateral pinwheel flap can be a practical multi-vector local fasciocutaneous option for selected scarred flank defects when adjacent tissue recruitment is feasible and a less morbid alternative to more extensive reconstruction is preferred. When the wound is complicated by osteomyelitis and colocutaneous fistula, however, durable healing depends on staged multidisciplinary management with source control, elimination of residual cavity, vascularized tissue support, NPWT, and appropriately timed delayed primary closure.

## Data Availability

The data presented in this study are available in this article.

## Abbreviations

The following abbreviations are used in this manuscript:

NPWT	Negative-Pressure Wound Therapy
